# Delayed-onset pancreatitis secondary to migration of a fully covered self-expanding metal biliary stent: a case report

**DOI:** 10.1093/jscr/rjag832

**Published:** 2026-09-20

**Authors:** Anupam K Gupta

**Affiliations:** Department of Surgery, Jackson Memorial Hospital, 1611 NW 12th Avenue, Miami, FL 33136, United States

**Keywords:** fully covered self-expanding metal stent, post-ERCP pancreatitis, biliary obstruction, stent migration, cholangitis, pancreatic duct obstruction, delayed-onset pancreatitis, malignant biliary stricture, case report, endoscopic retrograde cholangiopancreatography

## Abstract

Fully covered self-expanding metal stents (FCSEMS) are widely used for malignant biliary obstruction but may cause complications, including pancreatitis. Most cases occur shortly after placement, while delayed presentations are uncommon. We report a 74-year-old woman with metastatic adenocarcinoma and prior FCSEMS placement who presented months later with severe epigastric pain, nausea, vomiting, and markedly elevated lipase. Imaging showed an indwelling biliary stent without pancreatic abnormalities. Endoscopic retrograde cholangiopancreatography demonstrated migration of the FCSEMS into the ampulla with purulent biliary drainage. The migrated stent was removed and replaced, resulting in rapid symptom resolution. This case highlights FCSEMS migration as a rare cause of delayed pancreatitis due to mechanical obstruction of the pancreatic duct. Clinicians should consider this diagnosis in patients presenting with pancreatitis months after biliary stent placement, as prompt endoscopic intervention can lead to favorable outcomes.

## Introduction

The use of fully covered self-expanding metal stents (FCSEMS) has increased substantially for palliation of malignant biliary obstruction [1]. While FCSEMS offer superior patency compared to plastic stents, their placement carries a risk of complications, including acute pancreatitis. The mechanism is thought to involve radial compression or obstruction of the pancreatic duct by the stent [2]. Typically, post–endoscopic retrograde cholangiopancreatography (ERCP) pancreatitis occurs in the immediate postprocedural period [3–5]. We present an unusual case of delayed-onset pancreatitis occurring four months after FCSEMS placement, precipitated by stent migration to the ampulla and concurrent cholangitis.

## Case presentation

A 74-year-old African American woman with a history of hypertension, diabetes, hyperlipidemia, and metastatic adenocarcinoma to the spine underwent placement of a fully covered self-expanding metal stent (8 cm × 10 mm) for palliation of a mid-biliary stricture and obstructive jaundice 4 months prior. She presented to the emergency department with severe epigastric abdominal pain radiating to the back, accompanied by nausea and vomiting.

**Figure 1:**
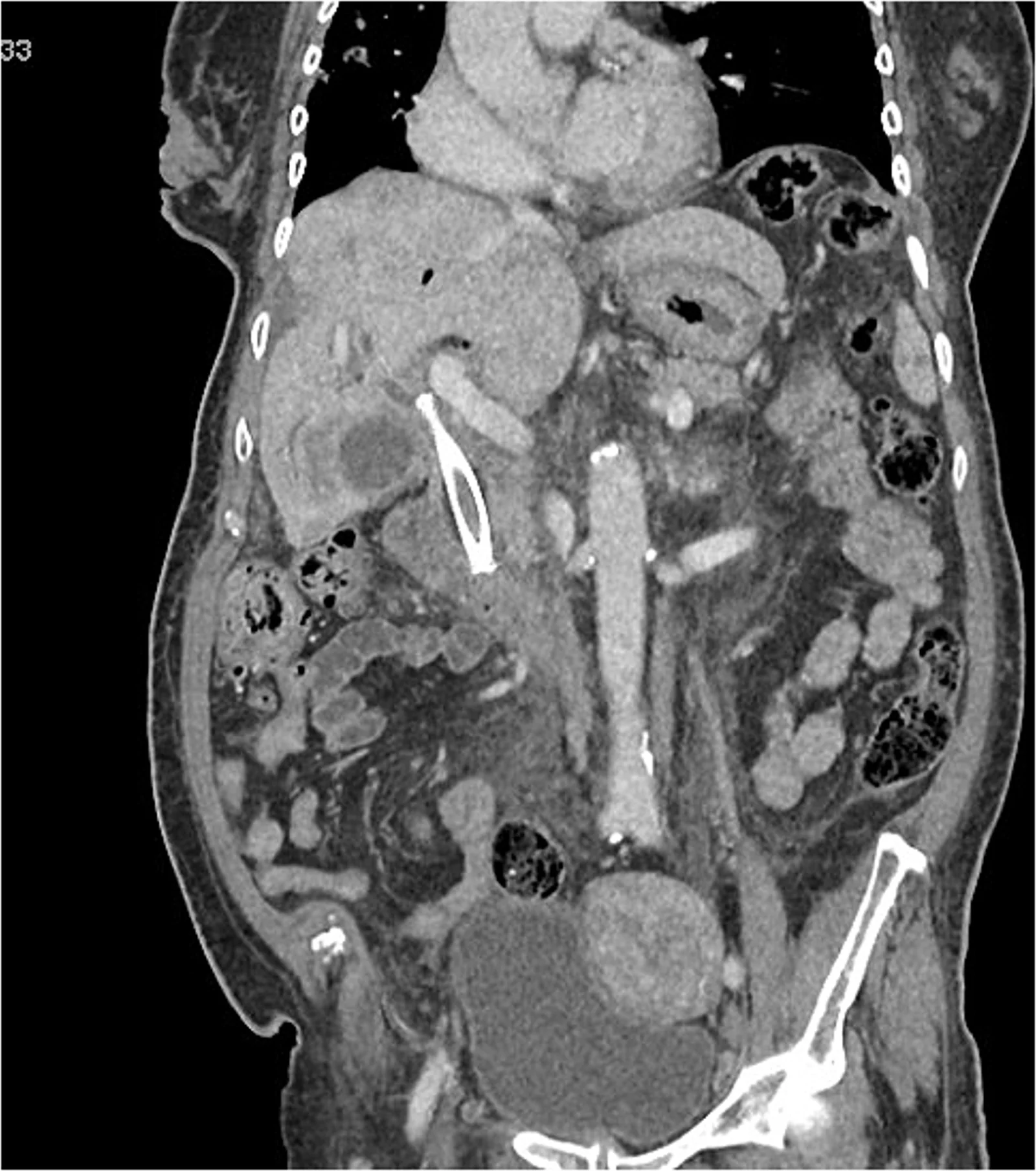
CT scan of the abdomen showing pneumobilia and an indwelling fully covered self-expanding metal stent in the biliary tree with maintained patency.

On examination, she was tender to deep palpation in the epigastrium. Laboratory testing showed elevated lipase (> 4000 U/L; reference 23–300 U/L) and leukocytosis (11.2 × 10^3^/μL). Liver function tests were mildly abnormal. Computed tomography (CT) demonstrated pneumobilia and an indwelling biliary stent (Fig. 1), without evidence of pancreatic inflammation or mass lesions.

The patient’s history, absence of alcohol use, and medication review revealed no alternative etiology for acute pancreatitis. Details regarding the prior biliary procedure and the make or model of the previously placed stent were not available to us. Imaging and endoscopic evaluation did not reveal any pancreatic pathology to explain the pancreatitis. She was managed conservatively with intravenous fluids and bowel rest. Despite initial improvement, she continued to have upper abdominal discomfort, prompting further evaluation with ERCP.

**Figure 2:**
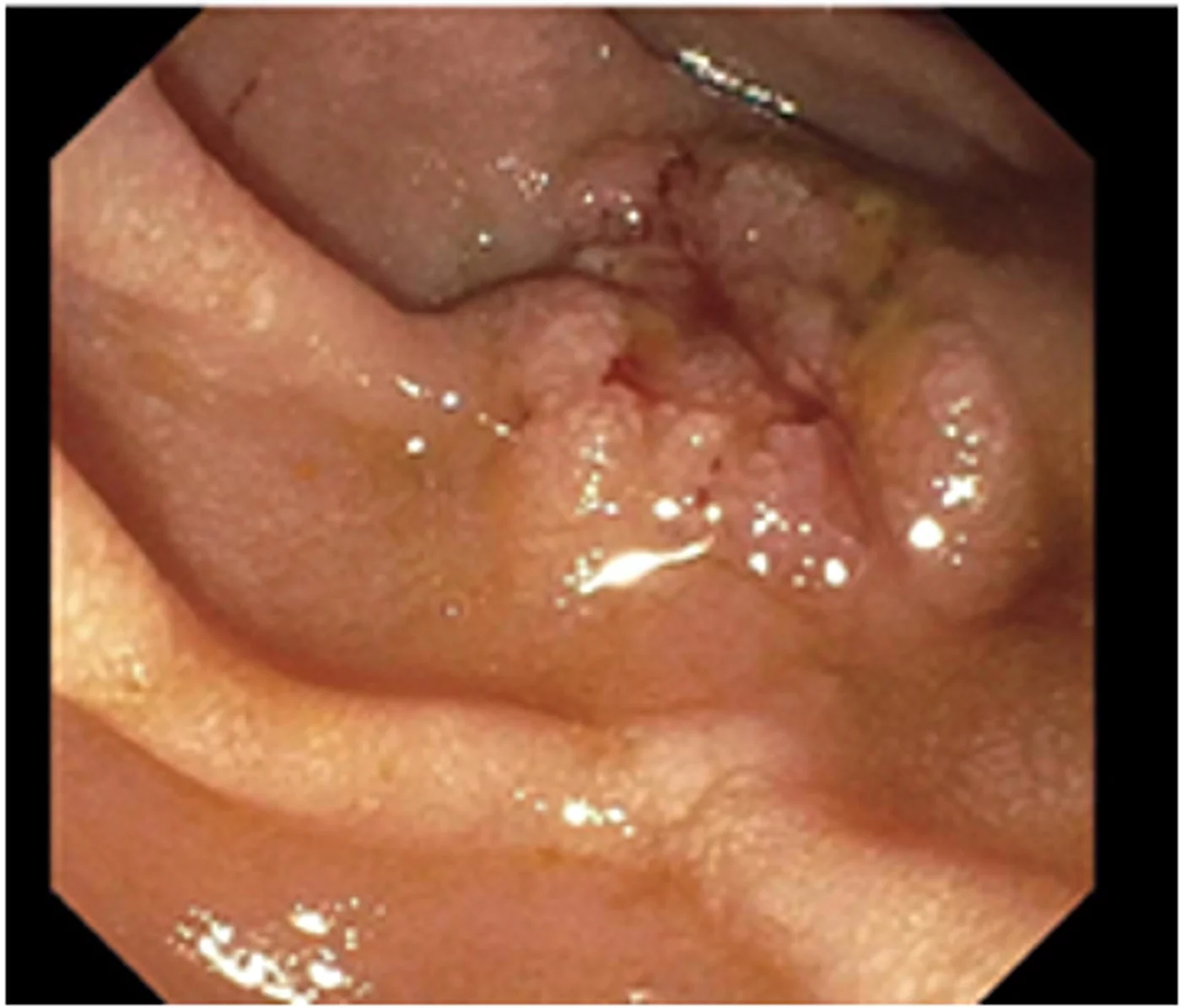
Endoscopic view showing the migrated FCSEMS partially embedded within the ampulla.

**Figure 3:**
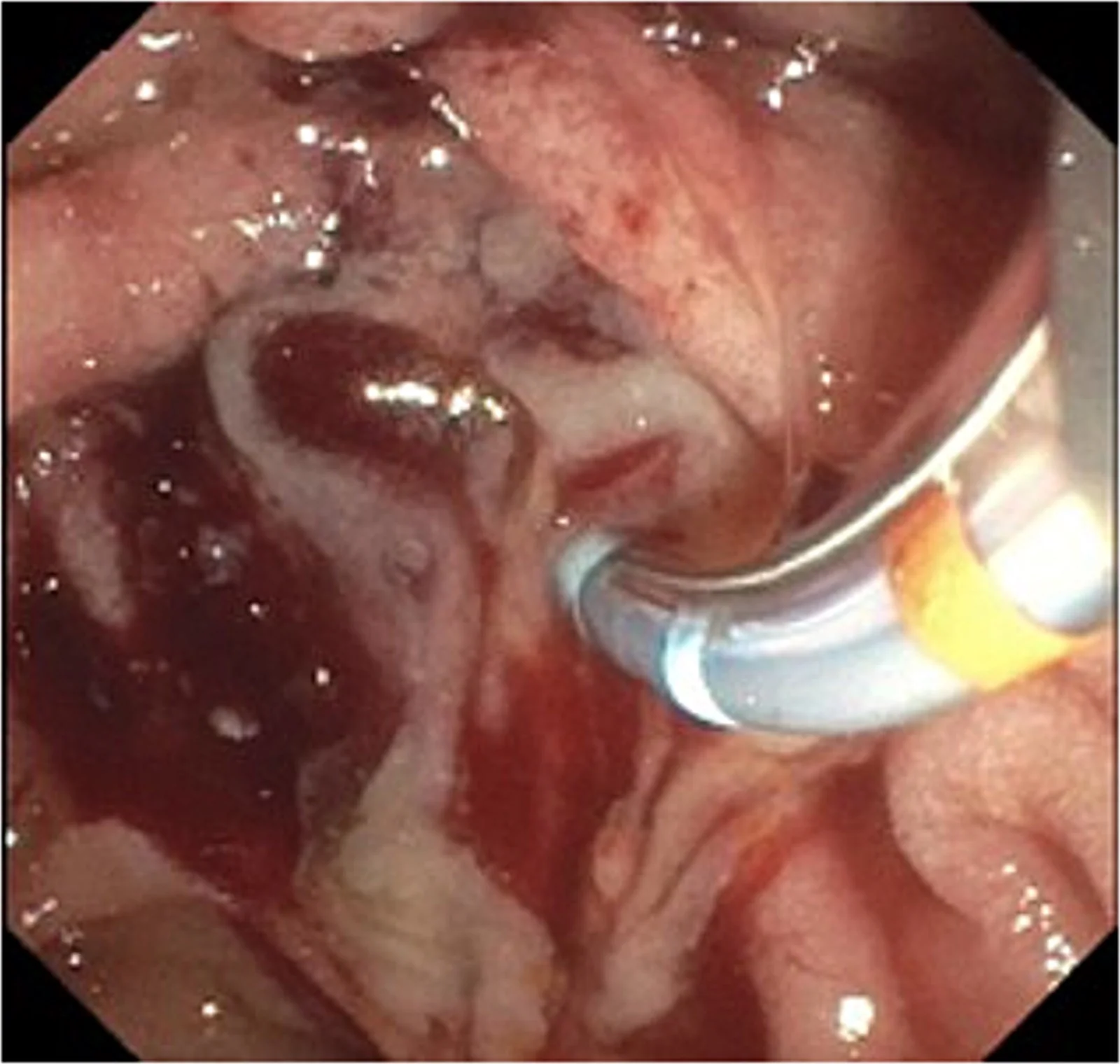
Purulent drainage observed from the biliary tree during stent removal.

ERCP revealed FCSEMS migration into the ampulla (Fig. 2) with purulent drainage from the biliary tree (Fig. 3). The original FCSEMS was removed, releasing a large volume of pus. Due to a high-grade mid-biliary stricture, a new fully covered self-expanding metal stent (6 cm × 10 mm) was placed, with ~1–2 cm extending into the duodenal lumen. The patient’s symptoms resolved rapidly, and she was discharged the following day.

## Discussion

Fully covered biliary self-expanding metal stents are considered standard of care for malignant biliary obstruction. However, pancreatitis is a recognized complication, occurring in 2%–14% of patients after transpapillary stent placement [5, 6]. The incidence is higher for metal stents than for plastic ones, due to the greater radial and axial forces exerted on the ductal systems [7].

Proposed mechanisms include direct compression of the pancreatic ductal orifice, activation of pancreatic enzymes due to ductal obstruction, and trauma during instrumentation [2, 5]. Pancreatitis has been noted more frequently in non-biliary malignancies, especially cholangiocarcinoma, metastatic lymphadenopathy, and ampullary tumors [5–8].

**Figure 4:**
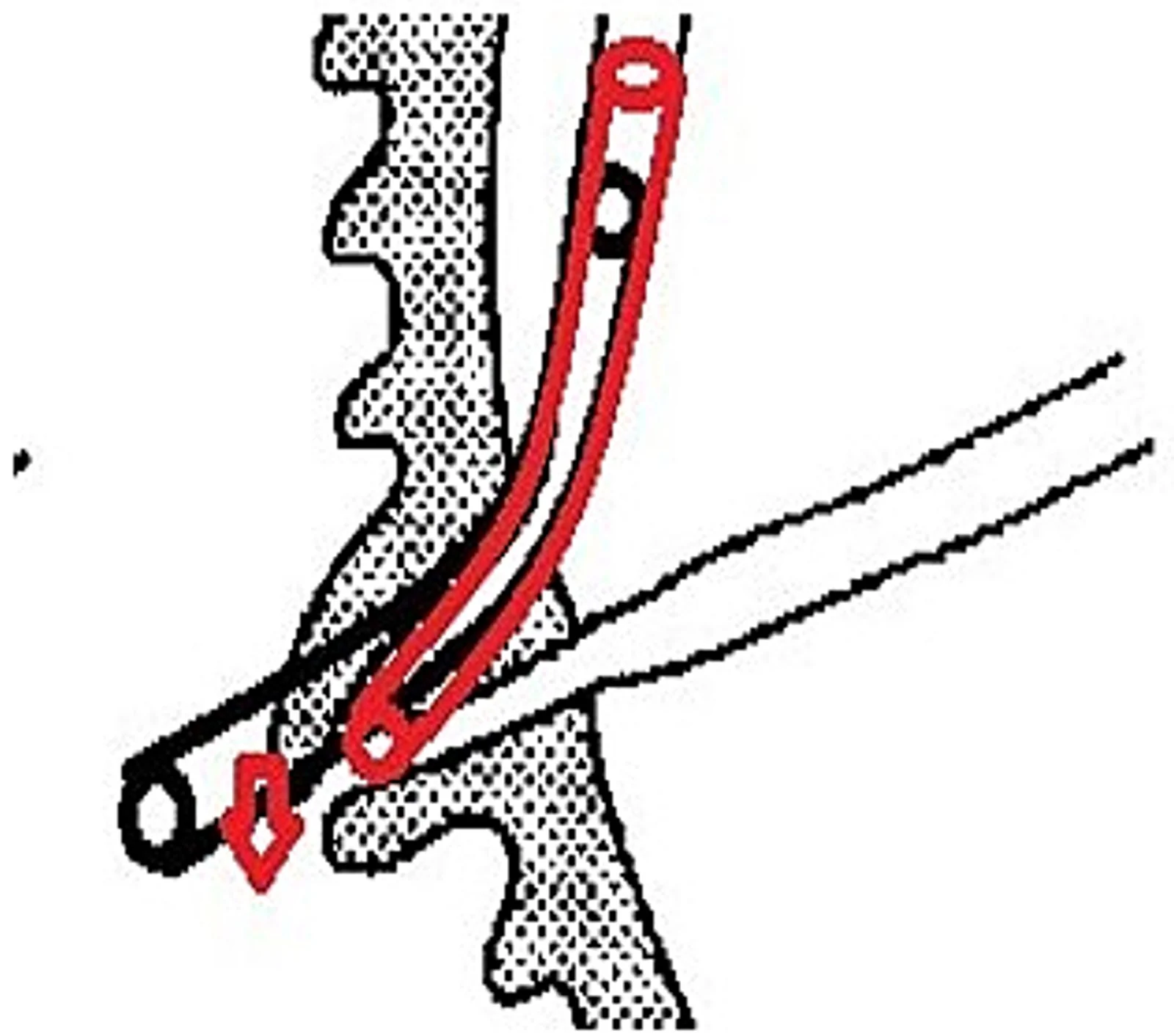
Diagram representation demonstrating medial deflection of the stent leading to obstruction of pancreatic ductal outflow.

In our case, migration of the FCSEMS to the ampulla resulted in medial deflection and partial obstruction of the pancreatic duct outflow (Fig. 4). This mechanical obstruction, compounded by localized inflammation from cholangitis, triggered acute pancreatitis months after initial stent placement. Removal and repositioning of the stent promptly relieved the obstruction and resolved symptoms.

## Conclusion

Fully covered self-expanding metal stents are invaluable for palliation of malignant biliary obstruction but are not without risk. Migration of a biliary stent to the ampulla can cause mechanical obstruction of the pancreatic duct, leading to delayed-onset pancreatitis. Clinicians should consider this possibility in patients presenting with pancreatitis months after FCSEMS placement. Careful imaging follow-up and prompt endoscopic management are crucial for favorable outcomes.
